# Living and deceased donor kidney transplant rates across Europe: ERA Registry Figure of the month

**DOI:** 10.1093/ckj/sfaf236

**Published:** 2025-08-06

**Authors:** Vianda S Stel, Alberto Ortiz, Anneke Kramer

**Affiliations:** ERA Registry, Department of Medical Informatics, Amsterdam UMC–Location University of Amsterdam, Amsterdam, the Netherlands; Amsterdam Public Health Research Institute, Quality of Care, Amsterdam, the Netherlands; Department of Nephrology and Hypertension, IIS-Fundacion Jimenez Diaz UAM, Madrid, Spain; Department of Medicine, Universidad Autonoma de Madrid, Madrid, Spain; ERA Registry, Department of Medical Informatics, Amsterdam UMC–Location University of Amsterdam, Amsterdam, the Netherlands; Amsterdam Public Health Research Institute, Quality of Care, Amsterdam, the Netherlands

**Figure 1:**
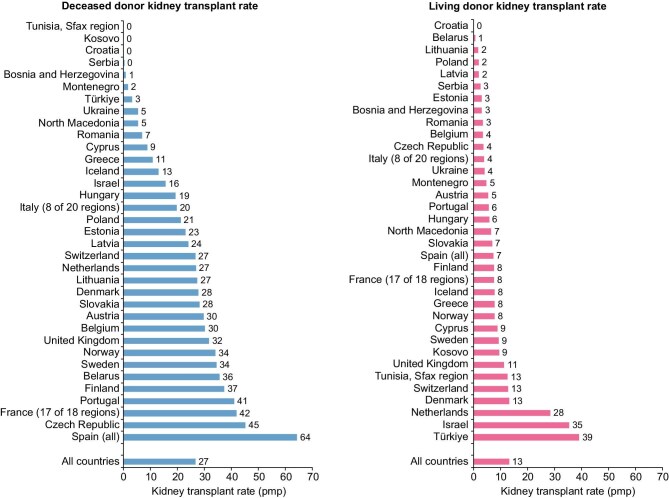
Kidney transplantations performed in 2022 per million population (pmp), by donor type and by country or region. **Source:** Boenink et al. NDT 2025, https://doi.org/10.1093/ckj/sfae405, Figure 11. The figure was slightly adapted to show differences at the level of the country and made no distinction in colours between countries providing individual or aggregated data to the ERA Registry. **Explanation:** Substantial differences exist in the living and deceased donor kidney transplantation (KT) rate within Europe in 2022. For all countries combined the deceased donor KT rate (27 pmp) was two-fold higher than the overall living donor KT rate (13 pmp). The deceased donor KT rate varied between 0 pmp in Tunisia (Sfax region), Kosovo, Croatia and Serbia, and 64 pmp in Spain. The living donor KT rate varied between 0 in Croatia and 39 pmp in Türkiye.

